# Absorbable Dermal Filler for Saddle Nose Deformity Associated With Wegener's Granulomatosis

**DOI:** 10.1111/jocd.70014

**Published:** 2025-02-07

**Authors:** Iris Alla, Felice Lorusso, Sergio Alexandre Gehrke, Sergio Rexhep Tari, Antonio Scarano

**Affiliations:** ^1^ Department of Innovative Technologies in Medicine and Dentistry University of Chieti‐Pescara Chieti Italy; ^2^ Department of Research, Bioface/PgO/UCAM, Montevideo, Uruguay. Department of Biotechnology Universidad Católica de Murcia (UCAM) Murcia Spain

**Keywords:** aesthetic medicine, dermal filler, rhinoplasty

Dear Editor,

Granulomatosis with polyangiitis, also known as Wegener granulomatosis (WG), is a necrotizing autoimmune disorder associated with vasculitis that could affect several different regions in humans, more frequently with multi‐organ inflammation of the kidneys, lungs, and respiratory tracts of Caucasian subjects [1, 2]. The head and neck implications range from 70% to 90% of the patients, with a higher frequency of nasal/paranasal, ear, and throat involvements with different grades of damage [1, 2, 3]. The most common clinical symptoms could include crusting, smell alterations, epistaxis, rhinorrhea, necrosis, septum perforation, sinusitis, and saddle‐nose deformity [4, 5, 6, 7, 8]. Saddle nose represents a clinical condition that could produce relevant functional and psychological distress with a decrease in the patient's quality‐of‐life [9, 10]. The granulomatosis with polyangiitis progression is able to sustain a chronic granulomatous inflammation able to determine a significant cartilage and facial bone basis resorption associated with soft tissues and mucosal structures changes [11]. The damage to the nasal and nasal sinuses could differ in severity grade, inflammation, and tissue thickening [3]. The loss of the cartilage and bone support is able to generate a significant collapse of the nasal framework, with the consequent requirement of a regenerative and grafting approach for the restoration of the natural profile [8, 11, 12]. The nasal framework is supported by a complex of vessel circulation [13] that should be considered in clinical practice. The main blood supply of the dorsal nose region is supported by the anterior ethmoid artery, while the lateral nose, ala, and the columella are supplied by the facial artery terminal arteries [13]. The most common complications of dermal filler treatment are pain, ulcerations, hypersensitivity, ecchymosis, swelling, graft migration [14], and nodule formation [15]. The learning curve requires a thorough understanding of the anatomical blood circulation variants and the intrinsic characteristics of the graft and the operative technique. We describe a clinical case of granulomatosis with polyangiitis saddle nose deformity treated with hyaluronic acid dermal filler for the restoration through a case report.

The patient presents a medical history of autoimmune granulomatosis with polyangiitis of over 25 years. From previous rheumatologic medical records, the patient was found to have oligoexpressed Wegener's granulomatosis, thus undergoing methotrexate administration. The tomography imaging of the paranasal sinuses shows modest thickening of the parietal mucosa of the maxillary sinuses, especially on the left, and some ethmoidal cells and the left sphenoidal sinus, hypertrophy of the left inferior turbinates. The last visit record of 2023 reported granulomatosis with polyangiitis, currently uncontrolled endonasal disease. The current pharmacological protocol included immunosuppressants, periodic nasal therapies including endonasal washes, endonasal decongestion sprays, mucolytics, and antibiotics. The nasal function and breathing were well maintained.

The patient therefore underwent an initial rhinofiller treatment on September 18, 2023. The region was disinfected and anesthetized through mepivacaine hydrochloride injection with no vasoconstrictor 30 mg/mL (Pierrel, Italy). Hyaluronic acid (Skin‐F 26, Italfarmacia, Rome, Italy) was injected into the collapsed nasal portion. The dermal filler used was a cross‐linked high molecular weight hyaluronic acid (26 mg/1 mL) specially containing glycine and proline. At all timepoints, the digital photographs have been analyzed measuring the following angle points: Subn‐Na‐Ri, Subn‐Na‐Su, Subn‐Na‐T, Pog‐Gl‐Ri, Pog‐Gl‐Su, and Pog‐Gl‐T (Figures 1 and 2). The nasal projection according to Goode have been calculated at the baseline, after the treatment, at 2 months and 4 months. The nasal tip projection according to Goode index have been calculated [16]. Following the treatment performed, the patient did not experience any postoperative complications other than redness and swelling of the area. From an aesthetic point of view, the first treatment provided initial support, which of course is only the first phase of the treatment to restore adequate nasal structure (Figure 3). Therefore, a second rhinofiller treatment was performed after 4 months using a cannula in order to perform a deeper correction with the hyaluronic acid injecting and to gain nasal support (Figure 4). At the end of treatment and after periodic checkups at 6 months, the patient appears to have a post‐operative period free of complications except for an initial redness that during healing disappeared completely. The patient has been undergoing treatment for 18 months and receives new hyaluronic acid injections approximately every 6 months.

**FIGURE 1:**
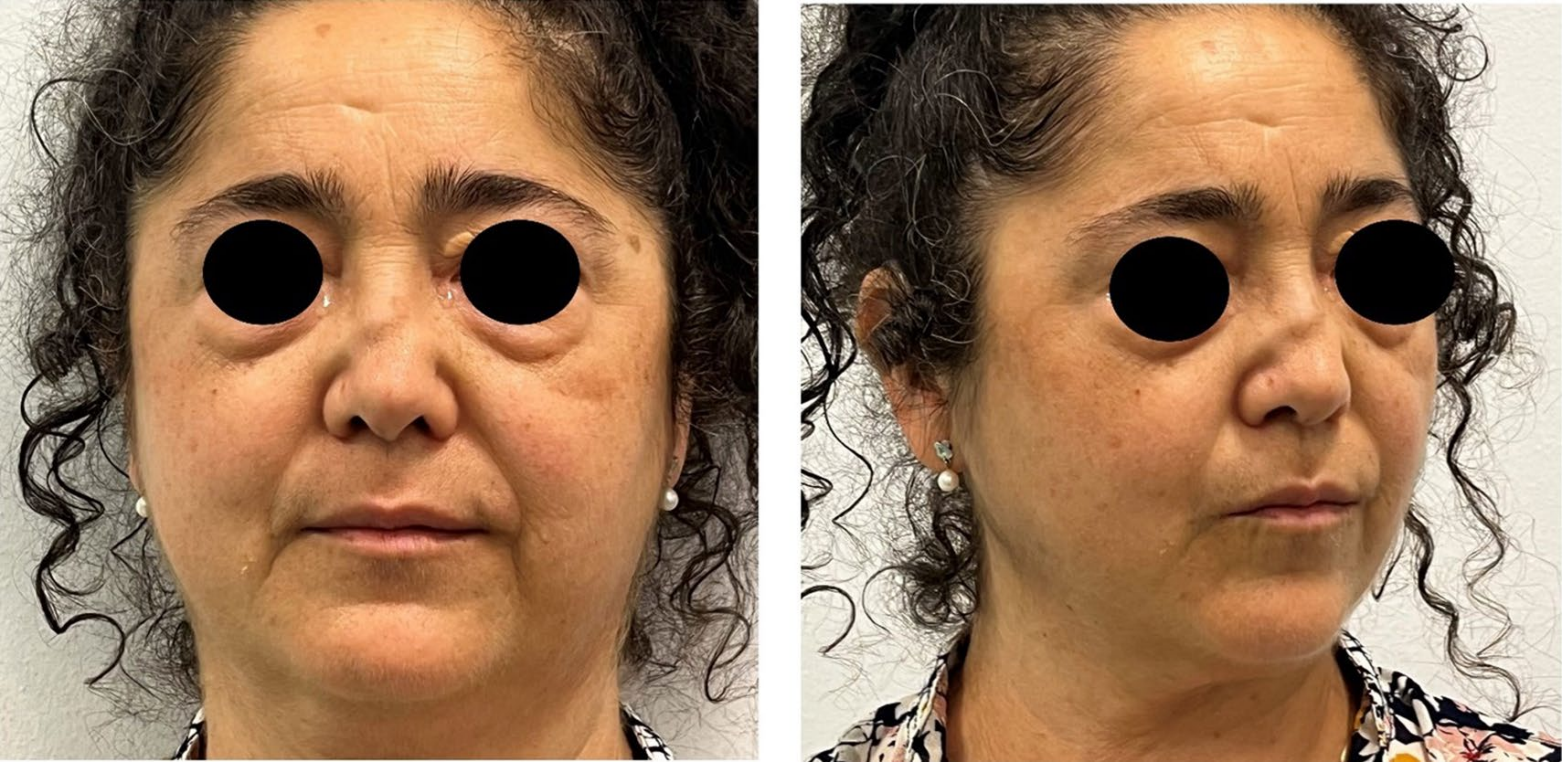
Clinical case at the baseline. A residual saddle nose deformity associated with WG with polyangiitis disease has been reported in the first visit.

**FIGURE 2:**
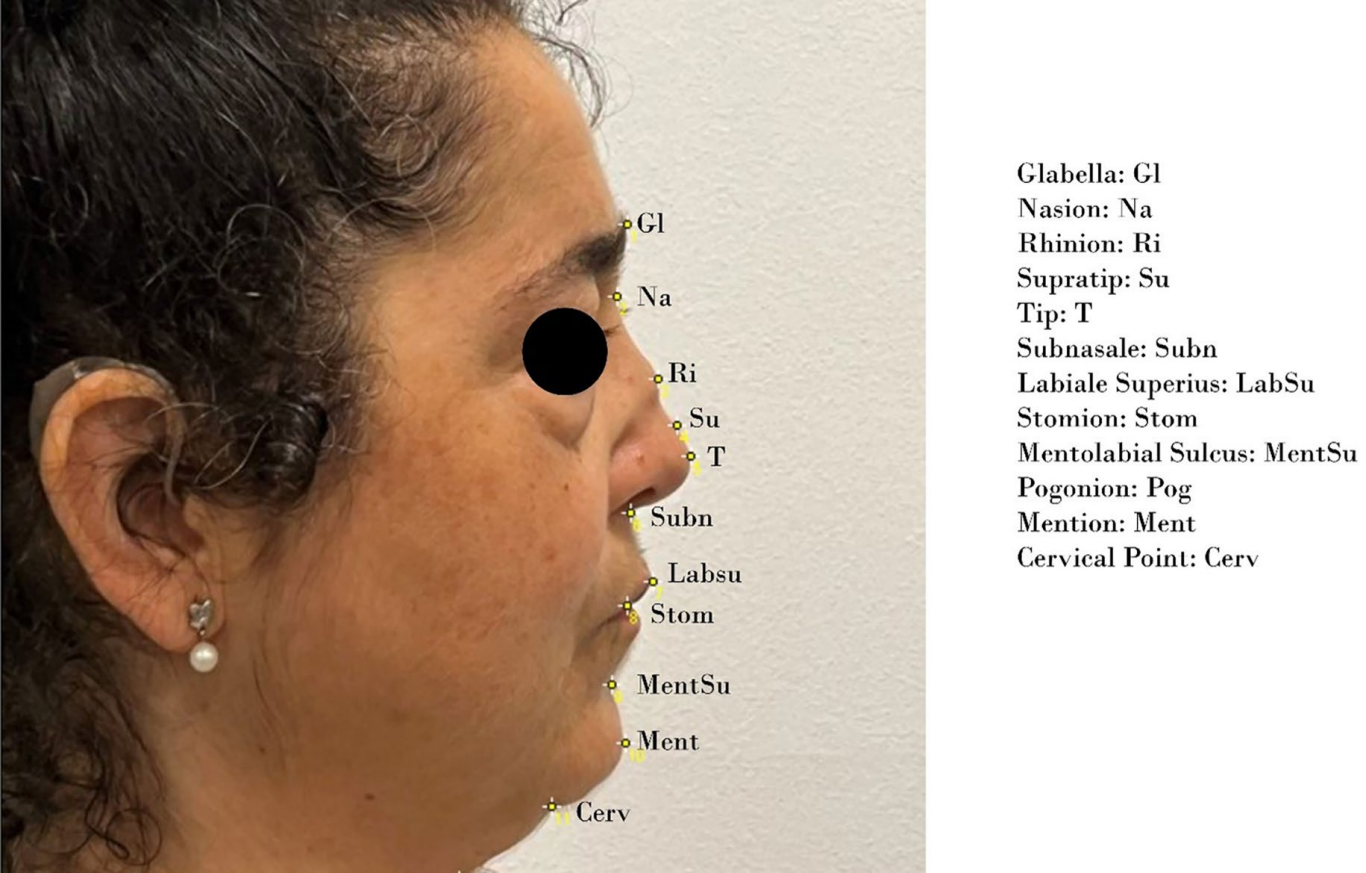
Synthesis of the facial mark points considered for the nasal profile analysis [Glabella: Gl; Nasion: Na; Rhinion: Ri; Supratip: Su; Tip: T; Subnasale: Subn; Labiale Superius: LabSu; Stomion: Stom; Mentolabial Sulcus: MentSu; Pogonion: Pog; Mention: Ment; Cervical Point: Cerv].

**FIGURE 3:**
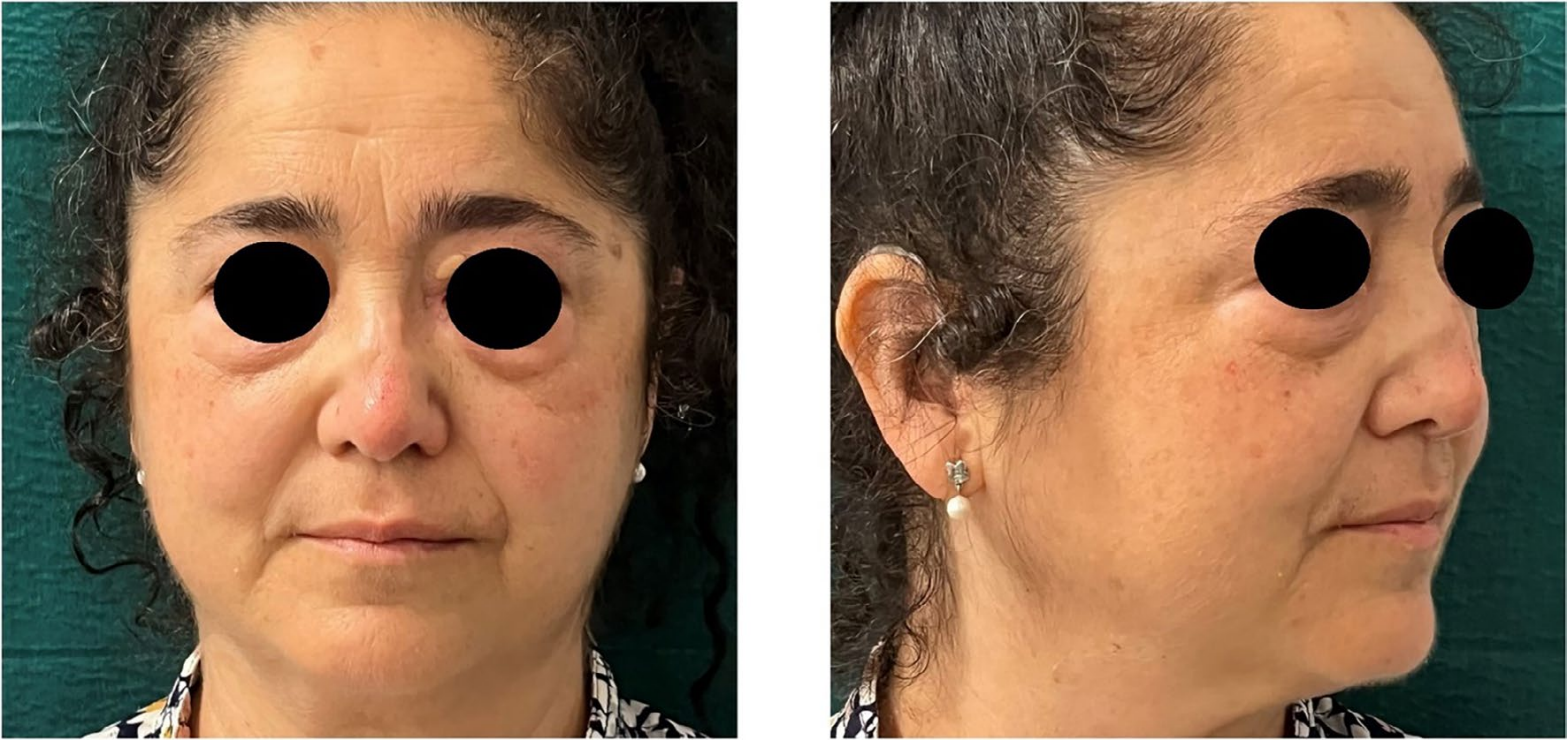
Nasal profile and appearance after the hyaluronic acid treatment.

**FIGURE 4:**
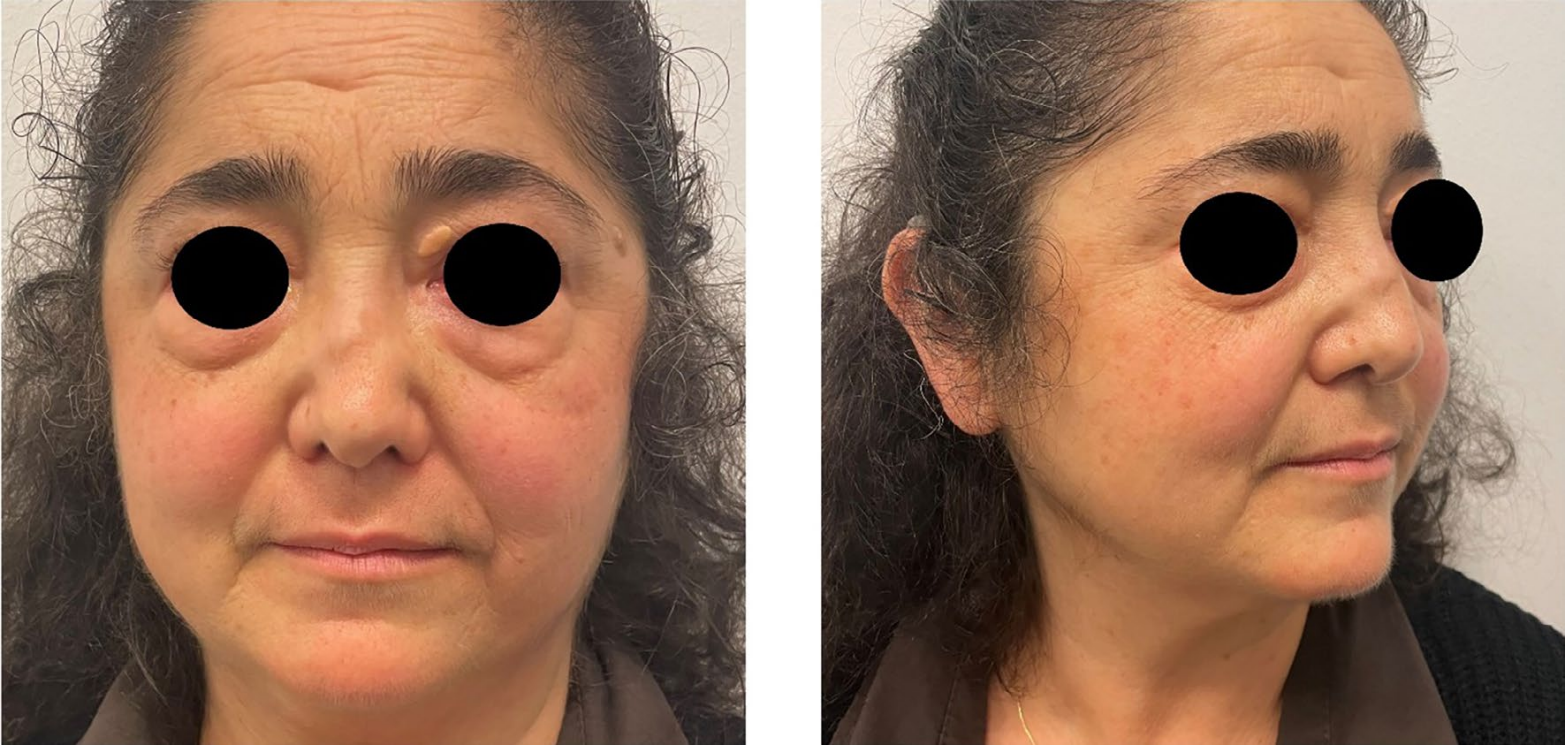
Nasal profile and appearance at 4 months from the treatment.

For reducing pain during infiltration of the filler, the nose area was covered with an anesthetic cream containing prilocaine and lidocaine for blocking nerve signals. The cream was applied 25–40 min before the treatment. Clamp the tip of the nose with the first and second fingers, insert the needle into the front of the columella, at 3 mm deep, and inject 0.1–0.3 mL of HA (average 0.22 mL) into the lower third of this space and withdraw the needle slightly before releasing using the Italian Technique [17]. A cannula was used to fill the supratip and rhinion. In December 2024, the patient underwent a follow‐up examination, during which no secondary issues related to the filler injection were observed. The check‐up confirmed that the initial treatment had been well‐tolerated and effective. At the patient's request, a new treatment with hyaluronic acid (HA) was performed. This additional session aimed to further enhance the aesthetic results and maintain the improvements achieved with the previous treatment (Figures 5 and 6).

**FIGURE 5:**
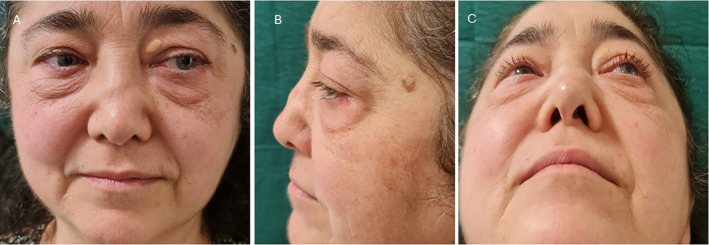
In the photos labeled A, B, and C, the nasal deformity is clearly visible before the new filler treatment.

**FIGURE 6:**
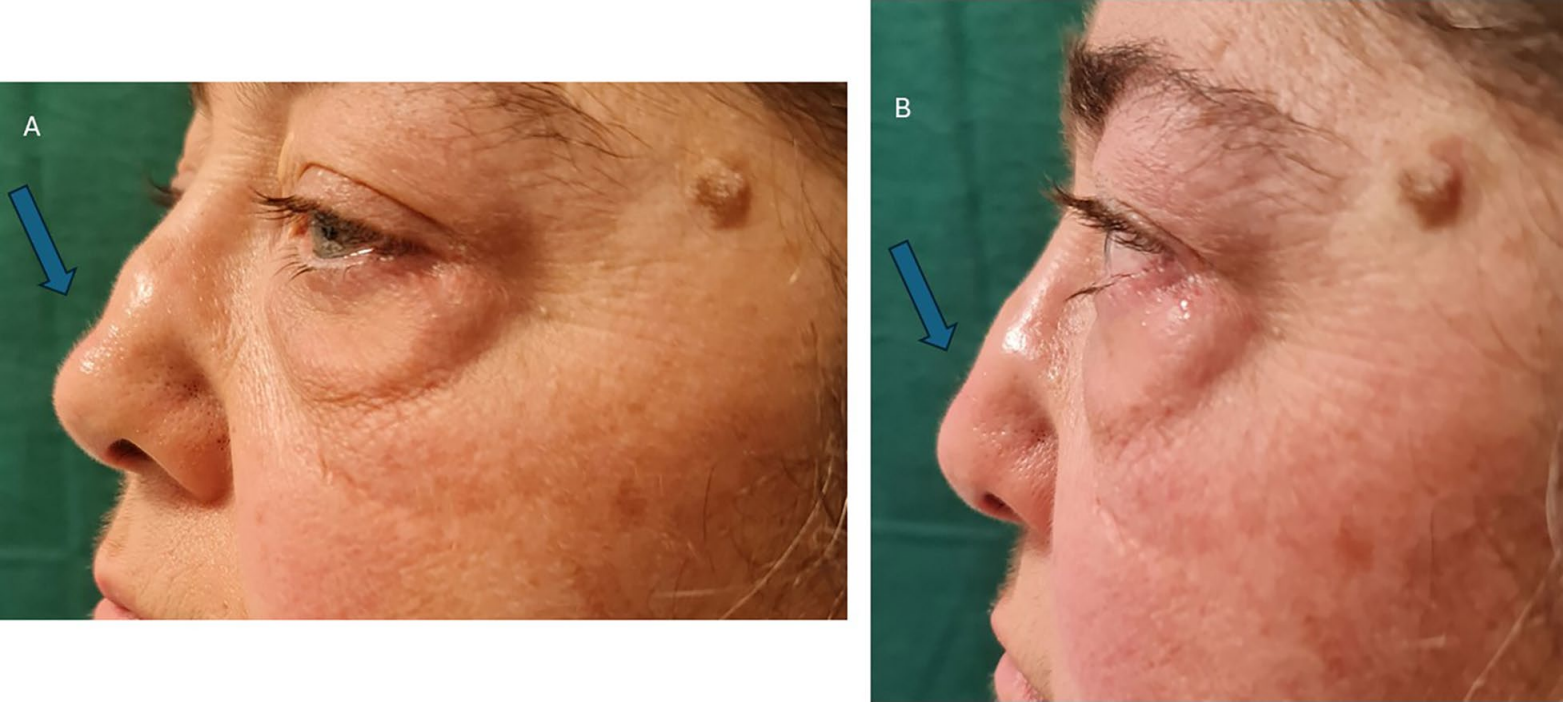
The difference between before and after filler injection is obvious (Arrows).

The present letter to the editor reported a rare case of saddle nose restoration with hyaluronic acid dermal filler. In general, the disease is not stable and the treatment setting of granulomatosis with polyangiitis involved nasal deformity requires an accurate multidisciplinary approach in respect to the status of the patient's illness associated with the systemic and local involvement [3], considering the clinical remission an elective criteria for the treatment [18].

In summary, the use of hyaluronic acid to correct nasal deformities in granulomatosis with polyangiitis is effective and safe (Figure 7).

**FIGURE 7:**
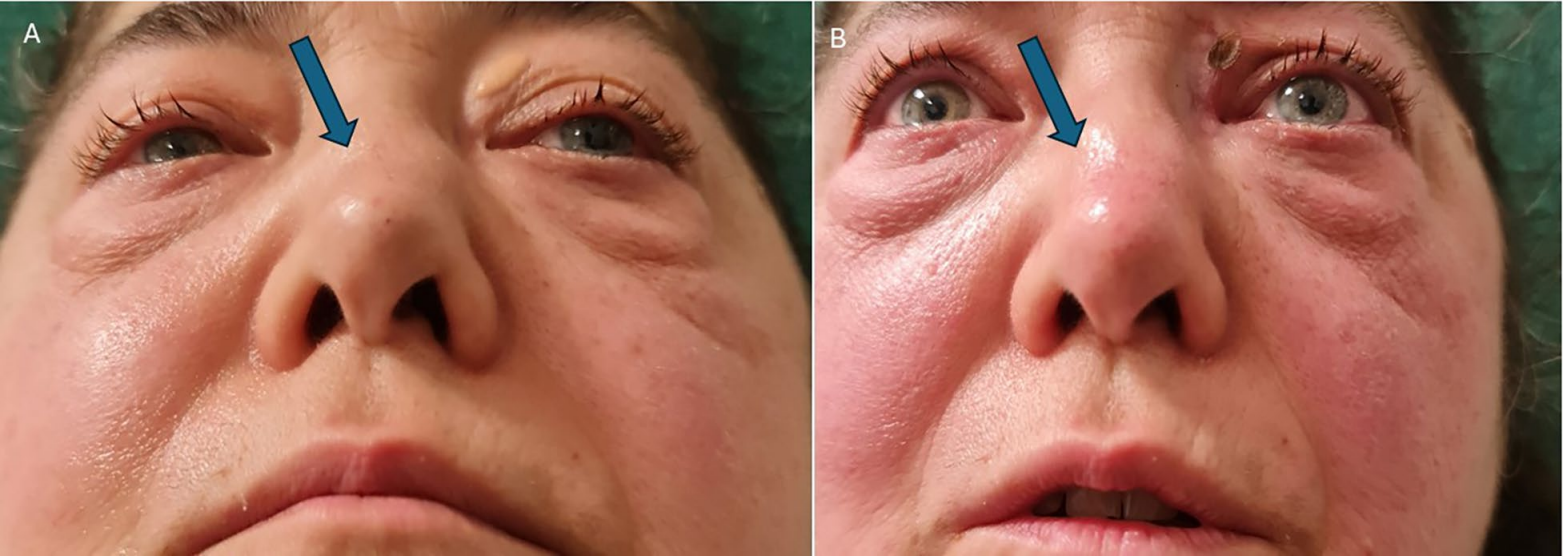
Also in this perspective, the difference before and after the filler injection is quite noticeable in photo B.

## Author Contributions

A.S., I.A., and S.R.T. performed the research. S.A.G., F.L., A.S., and S.R.T. designed the research study. A.S., I.A., and S.R.T. contributed essential reagents or tools. S.A.G., F.L., and A.S. analyzed the data. A.S. and F.L. wrote the paper.

## Ethics Statement

The investigation has been conducted in accordance with the Declaration of Helsinki and Good Clinical Practice Guidelines. The patients submitted the informed consent for the treatment and the anonymous data publication.

## Conflicts of Interest

The authors declare no conflicts of interest.

## Data Availability

The data that support the findings of this study are available from the corresponding author upon reasonable request.

## References

[1] F. Benoudiba , K. Marsot‐Dupuch , M. H. Rabia , et al., “Sinonasal Wegener's Granulomatosis: CT Characteristics,” Neuroradiology 45 (2003): 95–99, 10.1007/s00234-002-0885-9. 12592492

[2] J. Juri , N. Stiglmayer , and M. Mourits , “Review and New Insights on Wegener Granulomatosis,” Collegium Antropologicum 29, no. Suppl 1 (2005): 159–162. 16193702

[3] D. A. Cabral , A. G. Uribe , S. Benseler , et al., “Classification, Presentation, and Initial Treatment of Wegener's Granulomatosis in Childhood,” Arthritis and Rheumatism 60 (2009): 3413–3424, 10.1002/art.24876. 19877069

[4] M. J. Armstrong and A. H. Shikani , “Nasal Septal Necrosis Mimicking Wegener's Granulomatosis in a Cocaine Abuser,” Ear, Nose, & Throat Journal 75 (1996): 623–626. 8870370

[5] K. Holl‐Ulrich , M. Both , S. Gottschlich , W. L. Gross , P. M. Aries , and P. Lamprecht , “Clinical Images: Saddlenose Deformity Caused by Destructive Granulomatous Inflammation in Wegener's Granulomatosis,” Arthritis and Rheumatism 58 (2008): 834, 10.1002/art.23279. 18311791

[6] P. Burns , I. J. Keogh , K. Waheed , and C. V. I. Timon , “Wegener's Granulomatosis Masquerading as Unilateral Sinusitis,” Irish Medical Journal 97 (2004): 51. 15134271

[7] D. P. D'Cruz , E. Baguley , R. A. Asherson , and G. R. Hughes , “Ear, Nose, and Throat Symptoms in Subacute Wegener's Granulomatosis,” BMJ 299 (1989): 419–422, 10.1136/bmj.299.6696.419. 2506999 PMC1837272

[8] A. Gantous and R. F. Fernández‐Pellón Garcia , “Nasal Reconstruction in Granulomatosis With Polyangiitis: A Two Decade Review,” Facial Plastic Surgery & Aesthetic Medicine 25 (2023): 61–67, 10.1089/fpsam.2021.0348. 36044032 PMC9885542

[9] B. H. Ban , J. L. Crowe , and M. Tudor , “Saddle Nose Deformity and Granulomatosis With Polyangiitis,” QJM 111 (2018): 55, 10.1093/qjmed/hcx182. 29036610

[10] S. Chauhan and S. D'Cruz , “Images in Clinical Medicine. Saddle Nose Deformity,” New England Journal of Medicine 356 (2007): 2720, 10.1056/NEJMicm063822. 17596607

[11] N. Kesel , D. Köhler , L. Herich , et al., “Cartilage Destruction in Granulomatosis With Polyangiitis (Wegener's Granulomatosis) is Mediated by Human Fibroblasts After Transplantation Into Immunodeficient Mice,” American Journal of Pathology 180 (2012): 2144–2155, 10.1016/j.ajpath.2012.01.021. 22449947

[12] S. Kodama , N. Nomi , and M. Suzuki , “Wegener's Granulomatosis With Extensive Bone Abnormalities Mimicking Fungal Sinusitis,” Case Reports in Otolaryngology 2012 (2012): 103403, 10.1155/2012/103403. 22953096 PMC3420613

[13] S. DeVictor , A. A. Ong , and D. A. Sherris , “Complications Secondary to Nonsurgical Rhinoplasty: A Systematic Review and Meta‐Analysis,” Otolaryngology and Head and Neck Surgery 165 (2021): 611–616, 10.1177/0194599820987827. 33588622

[14] A. Scarano , F. Inchingolo , M. Di Carmine , et al., “Dermal Cosmetic Migration After Lip Augmentation Procedure: Clinical Management and Histological Analysis in a Case Report With Review of the Literature,” Surgeries 4 (2023): 223–234.

[15] S. Shahrabi‐Farahani , M. A. Lerman , V. Noonan , S. Kabani , and S. B. Woo , “Granulomatous Foreign Body Reaction to Dermal Cosmetic Fillers With Intraoral Migration,” Oral Surgery, Oral Medicine, Oral Pathology, Oral Radiology 117 (2014): 105–110, 10.1016/j.oooo.2013.10.008. 24332334

[16] D. M. Toriumi , D. G. Becker , and D. M. Cunning , Rhinoplasty Dissection Manual (Philadelphia, PA: Lippincott Williams & Wilkins, 1999).

[17] A. Scarano , A. Sbarbati , D. Amuso , R. Amore , S. R. Tari , and I. Alla , “The Use of Cross‐Linked Hyaluronic Acid in Non‐Surgical Rhinoplasty Using Italian Technique,” Aesthetic Plastic Surgery (2024), 10.1007/s00266-024-04197-6. Online ahead of Print. PMC11799021 38942953

[18] S. B. Cannady , P. S. Batra , C. Koening , et al., “Sinonasal Wegener Granulomatosis: A Single‐Institution Experience With 120 Cases,” Laryngoscope 119 (2009): 757–761, 10.1002/lary.20161. 19263410

